# Multilocus and interaction-based genome scan for alcoholism risk factors in Caucasian Americans: the COGA study

**DOI:** 10.1186/1471-2156-6-S1-S37

**Published:** 2005-12-30

**Authors:** Adrienne H Williams, W Mark Brown, Carl D Langefeld

**Affiliations:** 1 Section on Biostatistics, Department of Public Health Sciences, Wake Forest University Health Sciences, Medical Center Boulevard, Winston-Salem, NC 27157-1063, USA

## Abstract

In this paper, we applied the nonparametric linkage regression approach to the Caucasian genome scan data from the Collaborative Study on the Genetics of Alcoholism to search for regions of the genome that exhibit evidence for linkage to putative alcoholism-predisposing genes. The multipoint single-locus model identified four regions of the genome with LOD scores greater than one. These regions were on 7p near D7S1790 (LOD = 1.31), two regions on 7q near D7S1870 (LOD = 1.15) and D7S1799 (LOD = 1.13) and 21q near D21S1440 and D21S1446 (LOD = 1.78). Jointly modeling these loci provided stronger evidence for linkage in each of these regions (LOD = 1.58 on 7q11, LOD = 1.61 on 11q23, and LOD = 1.95 on 21q22). The evidence for linkage tended to increase among pedigrees with earlier mean age of onset at 8q23 (*p *= 0.0016), 14q21 (*p *= 0.0079), and 18p12 (*p *= 0.0021) and with later mean age of onset at 4q35 (*p *= 0.0067) and 9p22 (*p *= 0.0008).

## Background

The Collaborative Study on the Genetics of Alcoholism (COGA) is a study designed to identify the genetic influences of alcoholism. Although alcoholism itself and the corresponding risk factors are heritable, they are strongly believed to be complex genetic traits. Thus, in the search for genes that influence these traits we expect significant genetic heterogeneity, gene × gene, and gene × environment interactions. Statistical methods that have the flexibility to simultaneously consider multiple loci and environmental factors are potentially valuable tools in the search for putative disease-predisposing loci. The purpose of this paper is to examine the evidence for linkage using multilocus nonparametric linkage regression modeling and explore whether the evidence for linkage varies by the age of onset of alcoholism [1,2].

## Methods

The genotyped sample provided by COGA to the Genetics Analysis Workshop 14 consists of 102 Caucasian pedigrees (1,078 individuals) and 41 non-Caucasian pedigrees (526 individuals). Given the limited number of pedigrees of non-Caucasian ethnicity, this paper focuses on the self-reported Caucasian sample genotyped on 315 microsatellite markers and 15,406 autosomal single-nucleotide polymorphisms (SNPs). The alcohol dependence diagnosis required that an individual have DSM-III-R alcohol dependence and Feighner alc definite. This yielded four affection status classifications: 1) unaffected, 2) never drank, 3) unaffected with some symptoms, and 4) affected. The primary focus of these analyses will use affection status 4 only.

The initial genome scan linkage analyses were computed using the nonparametric linkage (NPL) (pairs) and NPL (all) statistics under 1) the exponential allele-sharing model implemented in GENEHUNTER PLUS [3] and 2) a conditional logistic regression parameterization denoted NPL regression [1,2]. This regression-based approach provides a one degree of freedom test of the evidence for linkage conditional on the evidence for linkage at the other loci in the model. Model building was performed using step-wise regression techniques. To test for an interaction between two loci, we included the two loci and their statistical interaction in the model and computed the one degree of freedom test of the interaction coefficient. In addition, we tested for interactions between the degree of sharing (identity by descent (IBD)) at a locus and the pedigree-specific mean age at alcoholism diagnosis. The *p*-value should be interpreted as a point-wise *p*-value and was not adjusted for the number of comparisons across the genome. All analyses are based on multipoint IBD estimates.

Ordered subset analyses (OSAs) [4] were computed to investigate the influence of a pedigree's mean age at alcoholism diagnosis on the evidence for linkage. Analyses were conducted ranking the mean family age of onset in ascending, and then in descending order. Linkage analyses were computed on contiguous subsets of pedigrees based on the mean age of onset ranking. The statistical significance of the change in the LOD score was evaluated by a permutation test under the null hypothesis that the ranking of the covariate is independent of the LOD score of the family on the target chromosome. Thus, the families were randomly permuted with respect to the covariate ranking and an analysis proceeded as above for each permutation of these data. The resulting empirical distribution of the change in the LOD score yielded a chromosome-wide *p*-value (Δ*p*). MERLIN [5] was also used to perform a genome scan and was subsequently used on the SNP data. Due to computation time only the chromosomes that showed linkage with the microsatellites were run through MERLIN for linkage analysis. Cox et al. [6] examined the decay of linkage disequilibrium (LD) across the genome and found little evidence that adjacent markers exhibited significant LD, thus validating the use of the SNP data for linkage analysis. More specifically, in the absence of parental genotype data LD between markers can inflate the type 1 error rate in linkage analysis. The allele frequencies in the MERLIN analyses were computed in MERLIN using founders. As above, multipoint IBD estimates were computed and the NPL regression analysis was computed based on the NPL (pairs) and NPL (all) statistics.

Using the strictest criteria of affection status, there were 643 affected individuals from 102 families. Among these families there were 656 relative pairs including: 404 full-sib pairs, 9 half-sib pairs, 8 grandparent-grandchild pairs, 178 avuncular pairs and 19 other relative pairs. The families consisted of pedigrees with two (*n *= 5), three (*n *= 30), four (*n *= 32), five (*n *= 21), six (*n *= 8) and seven or greater (*n *= 6) individuals diagnosed with alcoholism.

## Results

### Single-locus models

**Figure 1:**
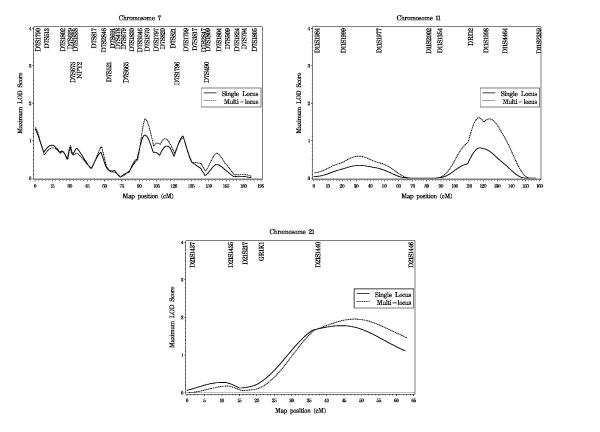
NPL single-locus and multiple locus regression.

Two chromosomes had maximum LOD scores greater than 1.0. Ordered by the magnitude of the LOD score, these regions were chromosome 21q22 (LOD = 1.78, 58 cM near D21S1440 and D21S1446), 7p21 (LOD = 1.31, 17 cM near D7S1790), 7q11 (LOD = 1.15, 112 cM nearest D7S1870), and 7q22 (LOD = 1.13, 145 cM near D7S1799). Chromosome 11q23 near D11S1998 (120 cM) provided modest evidence for linkage in the single-locus model (LOD = 0.81). Figure 1 displays the LOD score curves for the single-locus and multilocus analyses of chromosomes 7, 11, and 21. The linkage analysis of the SNP data using the NPL (pairs) statistic from MERLIN and NPL regression continued to provide evidence for linkage on chromosome 7p21 (LOD = 1.78) and 7q21 (LOD = 1.51) regions. However, the wide gap between microsatellite markers on chromosome 21 versus the high number of SNPs in this region led to differing results. Specifically, chromosome 21 no longer provided evidence for linkage to alcoholism. The information content for chromosome 21 went from an average across the chromosome of 60% to 94%. Chromosome 7 also had a marked increase in information content when moving from the SNP data to the microsatellite data, though the peaks remained comparably significant for this chromosome.

### Multilocus models

The results of the multilocus NPL regression model building using the microsatellite data are summarized in Table 1. The three peaks associated with chromosome 7q, 11q, and 21q continued to provide evidence for linkage. For all three positions in the model the conditional LOD score was larger and the interval of interest, as defined by the LOD-1 interval, was smaller than the corresponding single-locus LOD score and LOD-1 interval. Figure 1 displays the LOD score curves for each of these models. Interestingly, after adjusting for the evidence of linkage at chromosome 7 at 112 cM, the remaining regions of chromosome 7 no longer exhibited statistically significant evidence for linkage. There was not strong evidence of an interaction among these loci.

### Linkage and age of onset interaction analysis

Table 2 summarizes the results of the NPL regression interaction analysis with age of onset of alcoholism. The evidence for linkage tended to be stronger among pedigrees with an earlier mean age of onset of alcoholism at 8q23 (*p *= 0.0016), 14q21 (*p *= 0.0079), and 18q12 (*p *= 0.0021). Conversely, the evidence for linkage tended to be greater among those pedigrees with later age of onset of alcoholism at 4q35 (*p *= 0.0067) and 9p22 (*p *= 0.0008). The difference in mean age of onset tended to be about two to three years among those pedigrees that linked to these regions versus those that did not link (Table 2). None of the positions identified in the initial genome scan are among those regions that exhibited linkage evidence that varied by age of onset, and the positions identified in the interaction analysis with age of onset did not show significant evidence for linkage in the initial genome scan. The only possible exception was at 7q21 where pedigrees that had a greater mean age of onset tended to have increased evidence for linkage (*p *= 0.0350). A similar result was also found with OSA, subsetting on the families with the later age of onset of alcoholism increased the LOD score on 7q21 (LOD = 2.62 Δ*p *= 0.11). Subsetting on later age of onset yielded a significant increase in the LOD score on 17q23 (LOD = 2.01, Δ*p *= 0.0409). Subsetting on earlier mean age of onset only significantly increased the evidence for linkage at 9q33 (LOD = 1.72, Δ*p *= 0.0409). The result on 7p21 was within 10 cM of a similar result for earlier age of onset of alcoholism found with the NPL regression interaction analysis (*p *= 0.02586).

## Conclusion

Upon reviewing two previously published genome scans of alcohol dependence, two of our regions were identified in these published studies. The strongest evidence of linkage when looking across both the microsatellites and SNPs in this set of COGA data was on chromosome 7. The region on 7p was also identified in an American Indian population [8] within 10 cM of our peak with a nominal regression *p*-value of 0.009. The 7q peak is a series of peaks from about 100 cM to 160 cM; this region was found in the original COGA analyses [7] as well. The 11q peak was also replicated in the American Indian population [2] within 10 cM of our peak with a nominal regression *p*-value of 0.02. The 21q22 result showed up in both the ASM analysis and the NPL regression analysis, but completely disappeared when the SNP analysis was done.

Alcoholism is a genetically complex disease and therefore requires sophisticated consideration of multigenic and phenotypic influences. In this study methods that consider genetic heterogeneity, gene × gene interactions, gene × age-of-onset interactions, and joint modeling of multiple loci increased the evidence for linkage at three chromosomal locations, two of which had been previously identified as being associated with alcohol dependence. These methods reduced the linkage support interval at all three loci. In addition, testing for a dependence of the evidence for linkage on age of onset identified five additional regions of interest. These results suggest the potential utility of incorporating characteristics of complex genetic traits in the analysis.

## Abbreviations

COGA: Collaborative Study on the Genetics of Alcoholism

IBD: Identity by descent

LD: Linkage disequilibrium

NPL: Nonparametric linkage

OSA: Ordered subset analysis

SNP: Single-nucleotide polymorphism

## Authors' contributions

AHW, WMB and CDL contributed to the identification of the question and wrote the manuscript. AHW conducted all analyses.

## Figures and Tables

**Table 1:** Single- and multiple-locus NPL regression results

Chromosomal position	Single locus model			Multilocus model	
		
	Nearest marker	LOD	LOD-1 interval	LOD	LOD-1 interval
7p21 (17 cM)	D7S1790	1.31	pter-64		
7q11 (112 cM)	D7S1870	1.15	98–162	1.58	123–150
7q22 (145 cM)	D7S1799	1.13	96–163		
11q23(120 cM)	D11S1998	0.81	93–150	1.61	104–143
21q22 (58 cM)	D21S1440 / D21S1446	1.78	40-qter	1.95	43–77

**Table 2:** NPL regression interaction analyses for age at diagnosis

Chromosomal position	Nearest marker	Interaction *p*-value	Mean ± SD (*n*)	
			
			Linked	Unlinked
4q35 (216 cM)	D4S1652	0.00672	24.08 ± 5.26 (43)	21.63 ± 3.69 (59)
8q23 (139 cM)	D8S588	0.00164	21.26 ± 2.90 (34)	23.37 ± 5.08 (68)
9p22 (34 cM)	D9S925	0.00076	24.52 ± 5.40 (39)	21.52 ± 5.55 (63)
14q21 (49 cM)	D14S583	0.00793	21.32 ± 3.98 (39)	23.50 ± 4.73 (63)
18q12 (57 cM)	D18S877	0.00209	21.44 ± 3.90 (39)	23.43 ± 4.80 (63)
